# [5-Chloro-2-hydroxy-*N*′-(2-oxido­ben­zyl­idene)benzo­hy­dra­zidato]­pyridine­copper(II)

**DOI:** 10.1107/S1600536809020546

**Published:** 2009-06-06

**Authors:** Piyong Li, Dacheng Li, Xuefeng Shi

**Affiliations:** a College of Chemistry and Chemical Engineering, Liaocheng University, Shandong 252059, People’s Republic of China

## Abstract

In the title complex, [Cu(C_14_H_9_ClN_2_O_3_)(C_5_H_5_N)], the Cu^II^ ion exhibits a distorted *trans*-CuN_2_O_2_ square-planar geometry arising from the *O*,*O*,*N*-tridentate ligand and a pyridine mol­ecule. An intra­molecular O—H⋯N hydrogen bond occurs. In the crystal structure, weak inter­molecular C—H⋯π inter­actions generate a chain. The crystal studied was an inversion twin.

## Related literature

For background on the coordination chemistry of salicyl­aldehyde-type ligands, see: Bai *et al.* (2005 ▶). For information on C—H⋯π inter­actions, see: Nishio (2004 ▶).
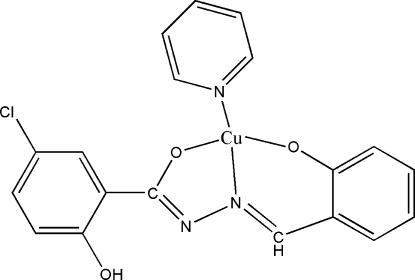


## Experimental

### 

#### Crystal data

[Cu(C_14_H_9_ClN_2_O_3_)(C_5_H_5_N)]
*M* *_r_* = 431.32
Monoclinic, 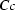

*a* = 23.586 (2) Å
*b* = 4.8268 (6) Å
*c* = 17.88540 (18) Å
β = 120.809 (2)°
*V* = 1748.8 (3) Å^3^
*Z* = 4
Mo *K*α radiation
μ = 1.43 mm^−1^
*T* = 298 K
0.39 × 0.28 × 0.17 mm

#### Data collection

Siemens SMART CCD diffractometer
Absorption correction: multi-scan (*SADABS*; Siemens, 1996 ▶) *T*_min_ = 0.606, *T*_max_ = 0.793
4087 measured reflections
2273 independent reflections
1849 reflections with *I* > 2σ(*I*)
*R*_int_ = 0.021

#### Refinement

*R*[*F* ^2^ > 2σ(*F* ^2^)] = 0.040
*wR*(*F* ^2^) = 0.116
*S* = 1.00
2273 reflections
244 parameters
2 restraints
H-atom parameters constrained
Δρ_max_ = 0.37 e Å^−3^
Δρ_min_ = −0.19 e Å^−3^
Absolute structure: Flack (1983 ▶), 725 Friedel pairs
Flack parameter: 0.50 (2)

### 

Data collection: *SMART* (Siemens, 1996 ▶); cell refinement: *SAINT* (Siemens, 1996 ▶); data reduction: *SAINT*; program(s) used to solve structure: *SHELXS97* (Sheldrick, 2008 ▶); program(s) used to refine structure: *SHELXL97* (Sheldrick, 2008 ▶); molecular graphics: *SHELXTL* (Sheldrick, 2008 ▶); software used to prepare material for publication: *SHELXTL*.

## Supplementary Material

Crystal structure: contains datablocks I, global. DOI: 10.1107/S1600536809020546/hb2989sup1.cif

Structure factors: contains datablocks I. DOI: 10.1107/S1600536809020546/hb2989Isup2.hkl

Additional supplementary materials:  crystallographic information; 3D view; checkCIF report

## Figures and Tables

**Table 1:** Selected bond lengths (Å)

Cu1—O3	1.897 (4)
Cu1—O1	1.934 (4)
Cu1—N2	1.945 (6)
Cu1—N3	1.965 (6)

**Table 2:** Hydrogen-bond geometry (Å, °)

*D*—H⋯*A*	*D*—H	H⋯*A*	*D*⋯*A*	*D*—H⋯*A*
O2—H2⋯N1	0.82	1.85	2.575 (9)	147
C16—H16⋯*Cg*1^i^	0.93	2.81	3.48 (3)	130

Symmetry code: (i) 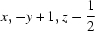. *Cg*1 is the centroid of the C9–C14 ring.

**Fig. 1.**
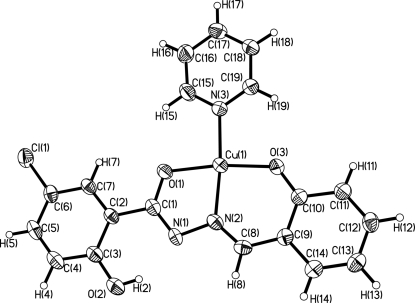
The molecular structure of (I), showing 40% probability displacement ellipsoids. H atoms have been omitted for clarity.

**Fig. 2.**
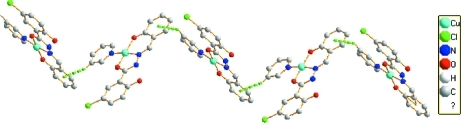
View of the chains in (I). Intermolecular C—H···π are shown as dashed lines. Most of H atoms are omitted.

## supplementary crystallographic information

### Comment

The chemistry of aroylhydrazones has gained a special attraction due to their coordination abilities to metal ions (Bai *et al.*,2005). However, researches on the complexes with salicylaldehyde-5-chlorosalicylichydrazone have not reported. So we have synthesized a new complex(Fig.1), which has been characterized by X-ray diffraction and elemental analysis. The structure of the title complex, (I), contains one ligand molecule, one pyridine molecule and one copper(II). The copper(II) coordination environment in the complex exhibits a distorted quadrilateral geometry (Table 1). In the crystal packing, the complex molecules are linked into one-dimensional chain by intermolecular C—H···π interactions (Nishio, 2004) (Table 2, Fig. 2).

### Experimental

A solution of salicylaldehyde (1.46 g, 12 mmol) in ethanol (10 ml) was added to a solution of 5-chlorosalicylichydrazine (1.87 g, 10 mmol) in ehanol (10 ml). The mixture was refluxed for 3 h, and then the precipitate was collected, washed several times with ethanol and dried *in vacuo* (yield 75.6%). m.p. > 300 K. A solution of Cu(OAc)_2_ (0.04 g, 0.2 mmol)in methanol (10 ml) was added to the mixture of salicylaldehyde -5-chlorosalicylichydrazone (0.058 g, 0.2 mmol)and sodium methylate (0.0324 g, 0.6 mmol) in pyridine (10 ml). A green solution was obtained after stirring for 4 h. After being filtrated, dimethyl ether was slowly diffused into the filtrate, then green blocks of (I) were obtained after several weeks (m.p. >400 K) Elemental analysis calculated for C_19_H_14_Cl_1_N_3_O_3_Cu_1_: C, 52.90; H, 3.27; N, 9.74. Found (%): C, 52.95; H, 3.19; N, 9.69

### Refinement

The H atoms were positioned with idealized geometry (C—H = 0.93Å, O—H = 0.82Å) and were refined as riding with *U*_iso_(H) = 1.2*U*_eq_(carrier).

### Crystal data

**Table:**

[Cu(C_14_H_9_ClN_2_O_3_)(C_5_H_5_N)]	*F*(000) = 876
*M**_r_* = 431.32	*D*_x_ = 1.638 Mg m^−^^3^
Monoclinic, *C**c*	Mo *K*α radiation, λ = 0.71073 Å
*a* = 23.586 (2) Å	Cell parameters from 1781 reflections
*b* = 4.8268 (6) Å	θ = 2.7–23.7°
*c* = 17.88540 (18) Å	µ = 1.43 mm^−^^1^
β = 120.809 (2)°	*T* = 298 K
*V* = 1748.8 (3) Å^3^	Block, green
*Z* = 4	0.39 × 0.28 × 0.17 mm

### Data collection

**Table:**

Siemens SMART CCD diffractometer	2273 independent reflections
Radiation source: fine-focus sealed tube	1849 reflections with *I* > 2σ(*I*)
graphite	*R*_int_ = 0.021
ω scans	θ_max_ = 25.0°, θ_min_ = 2.0°
Absorption correction: multi-scan (*SADABS*; Siemens, 1996)	*h* = −28→25
*T*_min_ = 0.606, *T*_max_ = 0.793	*k* = −5→5
4087 measured reflections	*l* = −17→21

### Refinement

**Table:**

Refinement on *F*^2^	Secondary atom site location: difference Fourier map
Least-squares matrix: full	Hydrogen site location: inferred from neighbouring sites
*R*[*F*^2^ > 2σ(*F*^2^)] = 0.040	H-atom parameters constrained
*wR*(*F*^2^) = 0.116	*w* = 1/[σ^2^(*F*_o_^2^) + (0.0729*P*)^2^ + 0.8658*P*] where *P* = (*F*_o_^2^ + 2*F*_c_^2^)/3
*S* = 1.00	(Δ/σ)_max_ < 0.001
2273 reflections	Δρ_max_ = 0.37 e Å^−^^3^
244 parameters	Δρ_min_ = −0.19 e Å^−^^3^
2 restraints	Absolute structure: Flack (1983), 725 Friedel pairs
Primary atom site location: structure-invariant direct methods	Flack parameter: 0.50 (2)

### Special details

**Table:**

Geometry. All e.s.d.'s (except the e.s.d. in the dihedral angle between two l.s. planes) are estimated using the full covariance matrix. The cell e.s.d.'s are taken into account individually in the estimation of e.s.d.'s in distances, angles and torsion angles; correlations between e.s.d.'s in cell parameters are only used when they are defined by crystal symmetry. An approximate (isotropic) treatment of cell e.s.d.'s is used for estimating e.s.d.'s involving l.s. planes.
Refinement. Refinement of *F*^2^ against ALL reflections. The weighted *R*-factor *wR* and goodness of fit *S* are based on *F*^2^, conventional *R*-factors *R* are based on *F*, with *F* set to zero for negative *F*^2^. The threshold expression of *F*^2^ > σ(*F*^2^) is used only for calculating *R*-factors(gt) *etc*. and is not relevant to the choice of reflections for refinement. *R*-factors based on *F*^2^ are statistically about twice as large as those based on *F*, and *R*- factors based on ALL data will be even larger.

### Fractional atomic coordinates and isotropic or equivalent isotropic displacement parameters (Å^2^)

**Table:**

	*x*	*y*	*z*	*U*_iso_*/*U*_eq_	
Cu1	0.18179 (4)	0.48947 (15)	0.25227 (4)	0.0455 (2)	
Cl1	0.00840 (11)	1.5179 (4)	−0.07019 (13)	0.0697 (5)	
N1	0.0517 (3)	0.6161 (12)	0.2113 (3)	0.0462 (13)	
N2	0.1052 (3)	0.4396 (12)	0.2649 (4)	0.0452 (14)	
N3	0.2566 (3)	0.5850 (12)	0.2378 (4)	0.0487 (13)	
O1	0.1235 (2)	0.7588 (9)	0.1676 (3)	0.0519 (11)	
O2	−0.0618 (2)	0.8534 (11)	0.1447 (4)	0.0721 (14)	
H2	−0.0319	0.7470	0.1764	0.108*	
O3	0.2275 (2)	0.1945 (8)	0.3304 (3)	0.0509 (11)	
C1	0.0675 (3)	0.7694 (13)	0.1643 (4)	0.0468 (15)	
C2	0.0182 (3)	0.9709 (11)	0.1036 (4)	0.0438 (14)	
C3	−0.0435 (4)	1.0041 (13)	0.0969 (5)	0.0545 (17)	
C4	−0.0874 (3)	1.1994 (15)	0.0388 (5)	0.064 (2)	
H4	−0.1282	1.2236	0.0343	0.076*	
C5	−0.0718 (3)	1.3556 (16)	−0.0116 (5)	0.0611 (19)	
H5	−0.1020	1.4840	−0.0503	0.073*	
C6	−0.0118 (3)	1.3239 (12)	−0.0053 (4)	0.0502 (15)	
C7	0.0333 (3)	1.1330 (13)	0.0522 (4)	0.0478 (15)	
H7	0.0740	1.1134	0.0564	0.057*	
C8	0.0986 (3)	0.2719 (14)	0.3155 (4)	0.0494 (16)	
H8	0.0590	0.2752	0.3150	0.059*	
C9	0.1478 (3)	0.0818 (12)	0.3722 (4)	0.0450 (15)	
C10	0.2086 (3)	0.0513 (12)	0.3749 (4)	0.0460 (15)	
C11	0.2519 (3)	−0.1564 (13)	0.4325 (5)	0.0539 (16)	
H11	0.2920	−0.1860	0.4357	0.065*	
C12	0.2365 (3)	−0.3133 (13)	0.4832 (4)	0.0576 (17)	
H12	0.2661	−0.4472	0.5197	0.069*	
C13	0.1771 (4)	−0.2753 (13)	0.4808 (4)	0.0579 (19)	
H13	0.1671	−0.3797	0.5162	0.069*	
C14	0.1341 (4)	−0.0825 (14)	0.4255 (5)	0.0540 (16)	
H14	0.0940	−0.0587	0.4230	0.065*	
C15	0.2503 (4)	0.7717 (17)	0.1806 (5)	0.069 (2)	
H15	0.2091	0.8520	0.1452	0.083*	
C16	0.3020 (4)	0.8541 (18)	0.1705 (6)	0.076 (2)	
H16	0.2958	0.9905	0.1302	0.092*	
C17	0.3626 (3)	0.7324 (16)	0.2205 (5)	0.0635 (18)	
H17	0.3981	0.7803	0.2143	0.076*	
C18	0.3687 (4)	0.5405 (17)	0.2790 (6)	0.080 (3)	
H18	0.4090	0.4535	0.3143	0.095*	
C19	0.3141 (4)	0.4733 (15)	0.2863 (6)	0.068 (2)	
H19	0.3193	0.3431	0.3277	0.082*	

### Atomic displacement parameters (Å^2^)

**Table:**

	*U*^11^	*U*^22^	*U*^33^	*U*^12^	*U*^13^	*U*^23^
Cu1	0.0378 (3)	0.0493 (4)	0.0461 (4)	0.0027 (3)	0.0192 (3)	−0.0002 (4)
Cl1	0.0771 (12)	0.0676 (12)	0.0674 (12)	0.0189 (9)	0.0392 (10)	0.0148 (9)
N1	0.039 (3)	0.044 (3)	0.046 (3)	0.012 (3)	0.015 (3)	0.000 (3)
N2	0.036 (3)	0.052 (3)	0.038 (3)	0.000 (3)	0.012 (3)	−0.010 (3)
N3	0.045 (3)	0.051 (3)	0.053 (4)	0.002 (3)	0.027 (3)	0.002 (3)
O1	0.041 (2)	0.061 (3)	0.053 (3)	0.007 (2)	0.024 (2)	0.008 (2)
O2	0.060 (3)	0.079 (3)	0.090 (4)	0.019 (3)	0.048 (3)	0.013 (3)
O3	0.045 (2)	0.046 (2)	0.060 (3)	0.007 (2)	0.026 (2)	0.005 (2)
C1	0.041 (3)	0.045 (3)	0.044 (4)	0.002 (3)	0.013 (3)	−0.010 (3)
C2	0.040 (3)	0.044 (3)	0.044 (3)	0.004 (3)	0.019 (3)	−0.011 (3)
C3	0.047 (4)	0.057 (4)	0.061 (4)	0.005 (3)	0.028 (3)	−0.004 (3)
C4	0.046 (3)	0.068 (5)	0.072 (5)	0.013 (3)	0.027 (4)	−0.006 (4)
C5	0.049 (4)	0.062 (4)	0.057 (5)	0.020 (4)	0.016 (3)	−0.003 (4)
C6	0.054 (4)	0.045 (4)	0.043 (4)	0.006 (3)	0.019 (3)	−0.006 (3)
C7	0.041 (3)	0.049 (4)	0.049 (4)	0.005 (3)	0.019 (3)	−0.012 (3)
C8	0.045 (3)	0.054 (4)	0.054 (4)	−0.004 (3)	0.030 (3)	−0.009 (3)
C9	0.053 (3)	0.035 (3)	0.048 (4)	−0.003 (3)	0.026 (3)	−0.010 (3)
C10	0.048 (3)	0.038 (3)	0.048 (4)	−0.006 (3)	0.021 (3)	−0.010 (3)
C11	0.054 (4)	0.040 (3)	0.065 (4)	−0.005 (3)	0.028 (3)	−0.006 (3)
C12	0.060 (4)	0.043 (4)	0.057 (4)	0.003 (3)	0.021 (3)	−0.001 (3)
C13	0.069 (4)	0.053 (4)	0.050 (5)	−0.013 (4)	0.028 (4)	−0.005 (3)
C14	0.060 (4)	0.051 (4)	0.053 (4)	−0.003 (3)	0.031 (3)	−0.003 (3)
C15	0.050 (4)	0.083 (5)	0.067 (5)	0.015 (4)	0.024 (4)	0.017 (4)
C16	0.078 (5)	0.083 (5)	0.084 (6)	0.018 (5)	0.053 (5)	0.031 (5)
C17	0.052 (4)	0.081 (5)	0.062 (4)	−0.001 (4)	0.031 (3)	0.000 (4)
C18	0.047 (4)	0.095 (6)	0.092 (6)	0.009 (4)	0.033 (4)	0.033 (5)
C19	0.049 (4)	0.074 (6)	0.072 (6)	0.002 (3)	0.024 (4)	0.023 (4)

### Geometric parameters (Å, °)

**Table:**

Cu1—O3	1.897 (4)	C7—H7	0.9300
Cu1—O1	1.934 (4)	C8—C9	1.419 (9)
Cu1—N2	1.945 (6)	C8—H8	0.9300
Cu1—N3	1.965 (6)	C9—C14	1.401 (10)
Cl1—C6	1.737 (7)	C9—C10	1.416 (9)
N1—C1	1.310 (9)	C10—C11	1.426 (10)
N1—N2	1.413 (8)	C11—C12	1.364 (10)
N2—C8	1.282 (9)	C11—H11	0.9300
N3—C19	1.296 (10)	C12—C13	1.393 (10)
N3—C15	1.313 (9)	C12—H12	0.9300
O1—C1	1.292 (7)	C13—C14	1.359 (9)
O2—C3	1.351 (9)	C13—H13	0.9300
O2—H2	0.8200	C14—H14	0.9300
O3—C10	1.293 (8)	C15—C16	1.380 (11)
C1—C2	1.478 (8)	C15—H15	0.9300
C2—C7	1.386 (10)	C16—C17	1.370 (10)
C2—C3	1.405 (10)	C16—H16	0.9300
C3—C4	1.393 (10)	C17—C18	1.349 (11)
C4—C5	1.362 (11)	C17—H17	0.9300
C4—H4	0.9300	C18—C19	1.396 (12)
C5—C6	1.370 (10)	C18—H18	0.9300
C5—H5	0.9300	C19—H19	0.9300
C6—C7	1.385 (9)		
			
O3—Cu1—O1	171.5 (2)	N2—C8—C9	124.1 (6)
O3—Cu1—N2	91.9 (2)	N2—C8—H8	117.9
O1—Cu1—N2	81.0 (2)	C9—C8—H8	117.9
O3—Cu1—N3	93.6 (2)	C14—C9—C10	120.1 (6)
O1—Cu1—N3	93.7 (2)	C14—C9—C8	117.5 (6)
N2—Cu1—N3	173.5 (3)	C10—C9—C8	122.4 (6)
C1—N1—N2	109.1 (5)	O3—C10—C9	125.8 (6)
C8—N2—N1	118.0 (6)	O3—C10—C11	118.4 (6)
C8—N2—Cu1	128.0 (5)	C9—C10—C11	115.8 (6)
N1—N2—Cu1	114.0 (4)	C12—C11—C10	122.4 (7)
C19—N3—C15	118.0 (7)	C12—C11—H11	118.8
C19—N3—Cu1	121.1 (6)	C10—C11—H11	118.8
C15—N3—Cu1	120.8 (5)	C11—C12—C13	120.8 (6)
C1—O1—Cu1	111.3 (4)	C11—C12—H12	119.6
C3—O2—H2	109.5	C13—C12—H12	119.6
C10—O3—Cu1	127.5 (4)	C14—C13—C12	118.5 (6)
O1—C1—N1	124.6 (5)	C14—C13—H13	120.7
O1—C1—C2	117.5 (6)	C12—C13—H13	120.7
N1—C1—C2	117.9 (5)	C13—C14—C9	122.4 (7)
C7—C2—C3	119.1 (6)	C13—C14—H14	118.8
C7—C2—C1	119.0 (6)	C9—C14—H14	118.8
C3—C2—C1	121.9 (6)	N3—C15—C16	123.0 (7)
O2—C3—C4	118.6 (7)	N3—C15—H15	118.5
O2—C3—C2	122.5 (6)	C16—C15—H15	118.5
C4—C3—C2	118.9 (7)	C17—C16—C15	119.1 (7)
C5—C4—C3	121.2 (7)	C17—C16—H16	120.4
C5—C4—H4	119.4	C15—C16—H16	120.4
C3—C4—H4	119.4	C18—C17—C16	117.6 (7)
C4—C5—C6	120.1 (6)	C18—C17—H17	121.2
C4—C5—H5	120.0	C16—C17—H17	121.2
C6—C5—H5	120.0	C17—C18—C19	119.6 (7)
C5—C6—C7	120.3 (7)	C17—C18—H18	120.2
C5—C6—Cl1	120.7 (5)	C19—C18—H18	120.2
C7—C6—Cl1	119.0 (5)	N3—C19—C18	122.7 (8)
C6—C7—C2	120.5 (6)	N3—C19—H19	118.7
C6—C7—H7	119.8	C18—C19—H19	118.7
C2—C7—H7	119.8		
			
C1—N1—N2—C8	−179.6 (6)	O1—C1—C2—C3	−178.1 (6)
C1—N1—N2—Cu1	1.2 (6)	N1—C1—C2—C3	1.3 (9)
O3—Cu1—N2—C8	4.3 (6)	C7—C2—C3—O2	179.9 (6)
O1—Cu1—N2—C8	179.6 (6)	C7—C2—C3—C4	−0.1 (9)
N3—Cu1—N2—C8	−143 (2)	C1—C2—C3—C4	179.5 (6)
O3—Cu1—N2—N1	−176.6 (4)	O2—C3—C4—C5	−179.4 (7)
O1—Cu1—N2—N1	−1.2 (4)	C4—C5—C6—Cl1	179.1 (6)
N3—Cu1—N2—N1	36 (3)	C5—C6—C7—C2	0.4 (9)
O3—Cu1—N3—C19	−9.1 (7)	Cl1—C6—C7—C2	−178.7 (5)
O1—Cu1—N3—C19	175.1 (7)	C3—C2—C7—C6	−0.4 (9)
N2—Cu1—N3—C19	139 (2)	C1—C2—C7—C6	−180.0 (5)
O3—Cu1—N3—C15	173.4 (6)	N1—N2—C8—C9	179.5 (5)
O1—Cu1—N3—C15	−2.5 (6)	N2—C8—C9—C14	178.1 (6)
N2—Cu1—N3—C15	−39 (3)	Cu1—O3—C10—C9	1.9 (9)
O3—Cu1—O1—C1	34.5 (18)	Cu1—O3—C10—C11	−177.8 (4)
N2—Cu1—O1—C1	1.0 (4)	C14—C9—C10—O3	−178.4 (6)
N3—Cu1—O1—C1	−175.1 (4)	C8—C9—C10—O3	2.9 (9)
O1—Cu1—O3—C10	−37.5 (19)	C14—C9—C10—C11	1.3 (8)
N2—Cu1—O3—C10	−4.5 (5)	C8—C9—C10—C11	−177.4 (6)
N3—Cu1—O3—C10	172.0 (5)	O3—C10—C11—C12	178.6 (6)
Cu1—O1—C1—N1	−0.6 (7)	C9—C10—C11—C12	−1.0 (9)
Cu1—O1—C1—C2	178.7 (4)	C8—C9—C14—C13	178.5 (6)
N2—N1—C1—O1	−0.4 (8)	Cu1—N3—C15—C16	177.2 (7)
N2—N1—C1—C2	−179.7 (5)	N3—C15—C16—C17	1.7 (14)
O1—C1—C2—C7	1.4 (8)	Cu1—N3—C19—C18	−178.7 (7)
N1—C1—C2—C7	−179.2 (6)		

### Hydrogen-bond geometry (Å, °)

**Table:**

*D*—H···*A*	*D*—H	H···*A*	*D*···*A*	*D*—H···*A*
O2—H2···N1	0.82	1.85	2.575 (9)	147
C16—H16···Cg1^i^	0.93	2.81	3.48 (3)	130

Symmetry codes: (i) *x*, −*y*+1, *z*−1/2.
